# Effect of Process Conditions on Microstructure, Damping Capacity, and Mechanical Properties of Mn-Cu Alloys

**DOI:** 10.3390/ma18184391

**Published:** 2025-09-20

**Authors:** Liyan Dong, Qiangsong Wang, Yuan Wu, Haofeng Xie, Junru Gao, Xinlu Chai, Kexing Song

**Affiliations:** 1 State Key Laboratory of Nonferrous Structural Materials, China GRINM Group Co., Ltd., Beijing 100088, China; dly2568@163.com (L.D.); thf8725@163.com (H.X.); zxlc0819@163.com (J.G.); jr519r@163.com (X.C.); 2 State Key Laboratory for Advanced Metals and Materials, University of Science and Technology Beijing, Beijing 100083, China; qwer05262024@163.com; 3 GRIMAT Engineering Institute Co., Ltd., Beijing 101407, China; 4 General Research Institute for Nonferrous Metals, Beijing 100088, China; 5 Henan Academy of Sciences, Zhengzhou 450018, China; kxsong@haust.edu.cn

**Keywords:** Mn-Cu alloy, process condition, microstructure, damping capacity, mechanical property

## Abstract

This study investigated the effects of four heat treatment processes on the microstructure, damping capacity, and mechanical properties of Mn-Cu alloys. The results indicated that the alloy did not undergo spinodal decomposition and twinning after solution treatment. After solution and aging treatment, the alloy underwent spinodal decomposition and formed Mn-rich regions, increasing the martensitic transformation temperature (M_s_), promoting martensitic transformation, forming twin boundaries, and enhancing damping capacity and mechanical properties. The cryogenic treatment and furnace cooling process facilitated the process, promoted the formation of twin boundaries, and improved damping capacity, and the degree of promotion by furnace cooling process was more significant. In addition, cryogenic treatment promoted grain refinement, increased dislocation density, improved strength, and facilitated the improvement of mechanical properties. This provided a reference for preparing high-damping Mn-Cu alloys with good comprehensive performance.

## 1. Introduction

Mn-Cu alloy is a widely used high-damping vibration reduction alloy with excellent vibration reduction performance. As a typical twin-type damping alloy, Mn-Cu alloy undergoes martensitic twinning after martensitic transformation. The movement between twin boundaries or between twin boundaries and parent phase absorbs vibration energy, making the alloy have high damping capacity [1,2,3,4,5,6].

The Mn-Cu alloys with high Mn content have excellent damping performance, but their mechanical properties are insufficient, which hinders their practical industrial application. Therefore, it is necessary to appropriately reduce the Mn content of Mn-Cu alloys, sacrifice a certain damping capacity, and balance the damping capacity and mechanical properties by adding alloying elements and heat treatment processes, and this has also become a research focus [7,8,9].

Vitek et al. [10] were the first to propose that Mn-Cu alloys underwent spinodal decomposition in the metastable solid solution region, leading to the formation of twin structures. Subsequently, related research became more extensive. Smith et al. [11] found that aging increased the martensitic transformation temperature (M_s_) of Mn-Cu alloys, and formed Mn-rich regions through spinodal decomposition. Martensitic transformation occurred during cooling, and twinning occurred under stress release. Zhong et al. [12] studied the damping capacity of M2052 alloy after different heat treatments and found that the damping capacity of the alloy was significantly improved after aging at 435 °C for 4 h. If Mn-Cu alloy is only subjected to solid solution treatment, the M_s_ is lower than room temperature, resulting in insufficient twinning formation and damping capacity [13,14]. Therefore, Mn-Cu alloys need to undergo solid solution treatment first, followed by aging treatment to increase the M_s_ of the alloy and promote the formation of twinning by utilizing the Mn-rich regions formed by spinodal decomposition, thereby improving the damping capacity [10,11,12,13,14,15,16,17].

The cooling process of Mn-Cu alloy after aging treatment also had significant differences in microstructure and properties [18,19]. Yin et al. [18] subjected the Mn_18_Cu_6_Ni_2_Fe to a solution treatment at 900 °C for 1 h, and then cooled it to room temperature at different rates. The results indicated that as the cooling rate decreased, the M_s_ of the alloy increased significantly. Yin et al. [19] studied the influence of twin microstructure on twin boundary damping behavior. Under furnace cooling conditions, conventional twin bands with similar widths were observed, while twin bands with intersecting tips appeared in the specimens treated with water quenching.

The combination of cryogenic treatment and traditional heat treatment processes would significantly affect the damping capacity and mechanical properties [20,21,22,23]. Ding et al. [23] showed that cryogenic treatment + aging treatment could effectively control the microstructure and phase composition of M2052 alloy, promote the precipitation of γ’ phase, and thus improve the mechanical and damping capacity of the alloy.

Based on the above research, this study designed and prepared Mn-Cu damping alloys with different heat treatment processes (solution treatment, aging treatment with different cooling processes, cryogenic treatment), aiming to investigate the effects of processes on microstructure, damping capacity, and mechanical properties, and to determine the key to twinning control to maximize the comprehensive performance of Mn-Cu damping alloys with low Mn contents. This study not only contributed to optimizing alloy design, but also provided theoretical support for the development of high-performance damping materials.

## 2. Materials and Methods

The raw materials were 99.99% high-purity cathode Cu, 99.99% pure Mn, 99.99% pure Fe, 99.99% pure Al, and 99.99% Ni Mn Cu-based ingots, which were prepared using vacuum induction melting technology in an argon atmosphere. The chemical composition of the Mn-Cu alloy, verified by inductively coupled plasma mass spectrometry (ICP-MS, PerkinElmer NexION, Waltham, MA, USA), is shown in Table 1.

**Figure 1:**
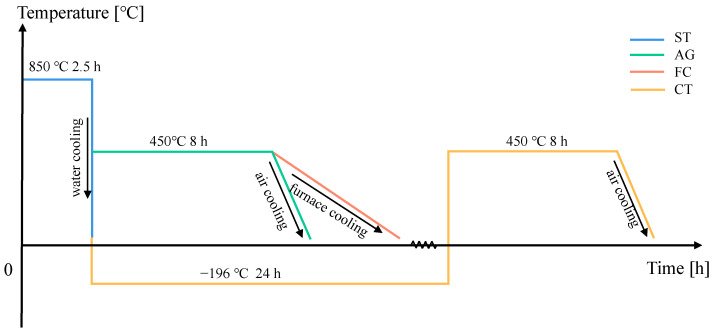
Four different heat treatment process timelines.

The prepared specimens were forged and hot-rolled to obtain rod-shaped specimens. After 2 h of solid solution treatment at 950 °C, the specimen continuously air-cooled to room temperature was labeled ST. After solid solution treatment and 2 h of aging treatment at 450 °C, the specimen was air-cooled or cooled in the furnace. The specimen was continuously air-cooled to room temperature and labeled AG, and the specimen cooled in the furnace was labeled FC. After solid solution treatment, it was first subjected to cryogenic technology (immersed in −196 °C liquid nitrogen for 24 h), then heated to room temperature, and finally aged at 450 °C for 2 h, and the specimen that was continuously air-cooled to room temperature and labeled CT. The detailed heat treatment process is shown in Table 2 and Figure 1.

After grinding and polishing, the microstructures of the specimens were observed and analyzed by energy spectrum using a scanning electron microscope (SEM, Zeiss, Oberkochen, Germany). The acceleration voltage of the scanning electron microscope was 15 kV, and the working distance was 10 mm. The chemical composition was analyzed using an energy dispersive X-Ray spectrometer (EDS, Oxford Instruments, Abingdon, Oxfordshire, UK).

The microstructures of the specimens were characterized using transmission electron microscopy (TEM, FEI, Hillsboro, OR, USA). In order to meet the requirements of TEM analysis, the specimens were ground and polished using silicon carbide paper, and thinned to a thickness of approximately 100 to 150 mm. Subsequently, the ion slicer (JEOL, Tokyo, Japan) was used to perform ion milling on the flakes to improve electronic transparency. TEM analysis was conducted under high-vacuum conditions at 10^−5^–10^−6^ to ensure optimal imaging quality. We analyzed the crystal structure of the specimens using X-Ray diffraction (XRD, SmartLab, Rigaku Corporation, Akishima, Japan).

During the testing process, the specimens underwent diffraction scanning at a speed of 3°/min within an angle range of 20° to 90° to obtain accurate crystallographic information. The mechanical properties were tested using a universal testing machine (WDW-300, Changchun Kexin Testing Machine Co., Ltd., Changchun, China), with a strain rate set to 0.0012 s^−1^. The samples of the standard tensile specimen (M6 mm), with a parallel segment length of 35 mm and a diameter of 3 mm. To reduce errors, three tensile samples were selected for testing in each group, and the average values of tensile strength, yield strength, and elongation were recorded. To evaluate the damping capacity (Q^−1^), a dynamic mechanical thermal analyzer (DMA, Waters Corporation, Milford, MA, USA) was used for testing, and the sample was prepared in the following dimensions: 30 mm × 7 mm × 1.5 mm. The sample was tested at room temperature with a testing frequency of 1 Hz, and the strain amplitude increased from 0 to 1 × 10^−3^.

## 3. Results

**Figure 2:**
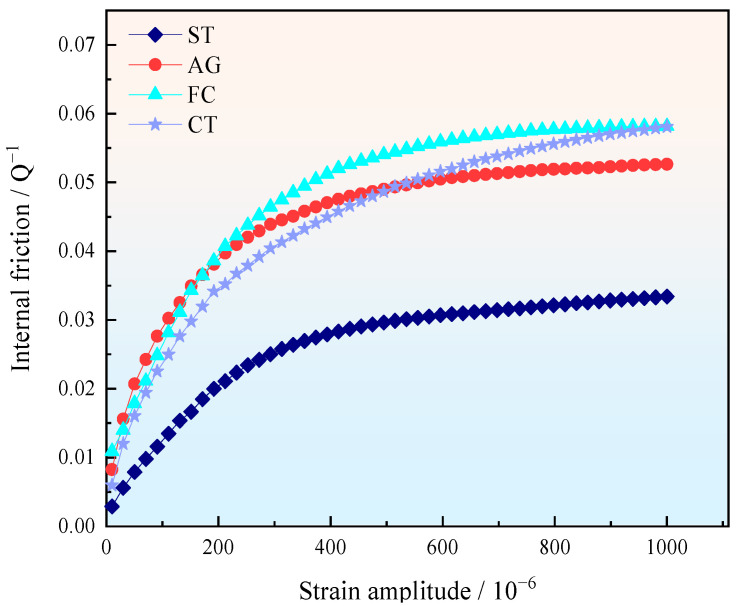
The variation curve of the damping capacity of the specimens with the strain amplitude.

The variation curves of internal friction values of different specimens with strain amplitude at room temperature (frequency of 1 Hz) are shown in Figure 2. From the graph, it can be seen that as the strain amplitude increased, the internal friction values of these four specimens showed a trend of rapid increase followed by stabilization. According to the graph, the internal friction values of the specimens were calculated at a strain amplitude of 1 × 10^−3^. The internal friction value of the ST specimen was 0.033. After aging treatment, the internal friction value increased significantly, reaching 0.053 for the AG specimen. The internal friction values of FC and CT were the same, both at 0.058, respectively, indicating that cryogenic treatment and furnace cooling have a promoting effect on improving damping capacity.

**Figure 3:**
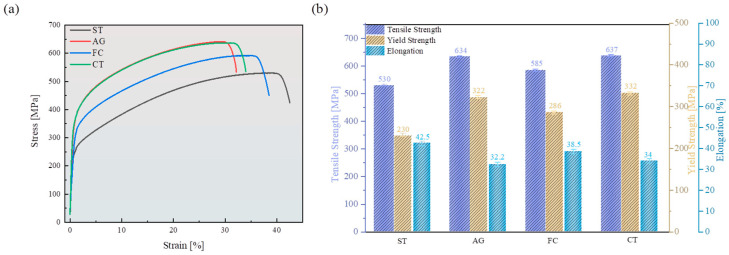
Mechanical properties of Mn-Cu alloy: (**a**) stress–strain curve; (**b**) comparison of mechanical properties.

The stress–strain curves of different specimens at room temperature are shown in Figure 3. Through comparison, it was found that the ST specimen had the highest elongation but the lowest yield strength. The strength of AG and CT specimens was similar, but the elongation of the CT specimen was significantly better than that of the AG specimen. The strength and plasticity of the FC specimen were at the intermediate level for the four specimens. Overall, on the basis of solid solution treatment, the aged specimens had improved their strength. Compared to air-cooling after aging treatment, furnace cooling increased plasticity but slightly reduced strength. Cryogenic treatment promoted the improvement of plasticity while maintaining the same strength.

**Figure 4:**
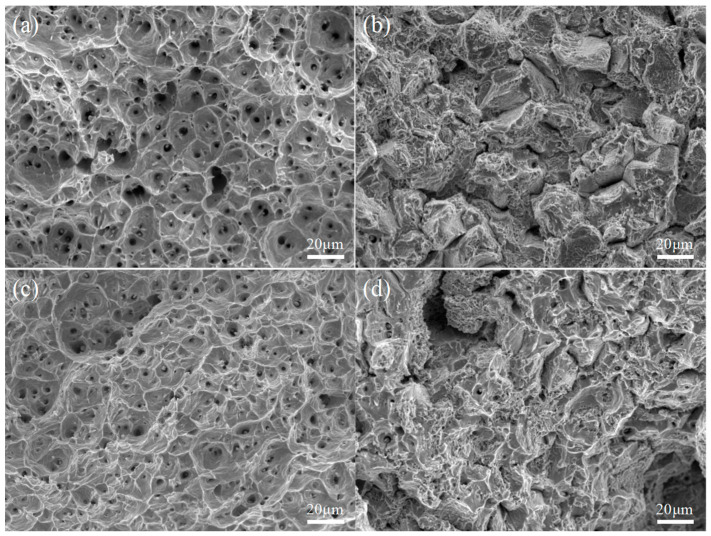
Tensile fracture morphology of specimens subjected to different process conditions: (**a**) ST; (**b**) AG; (**c**) FC; (**d**) CT.

The tensile fracture of the specimens was shown in Figure 4, where it was observed that the fracture of the ST and FC specimens exhibited honeycomb-like dimples, which were typical ductile fractures. Comparison showed that the ST specimen had the largest dimple size, followed by the FC specimen. The fracture of the CT and AG specimens had cleavage steps and only a small portion of ductile dimples, indicating that the fracture modes of the CT and AG specimens were cleavage fracture and ductile fracture. The size and number of ductile dimples in the CT specimen were larger than those in the AG specimen. The fracture morphology of the specimen further confirms the plastic deformation of the tensile curve in Figure 3.

**Figure 5:**
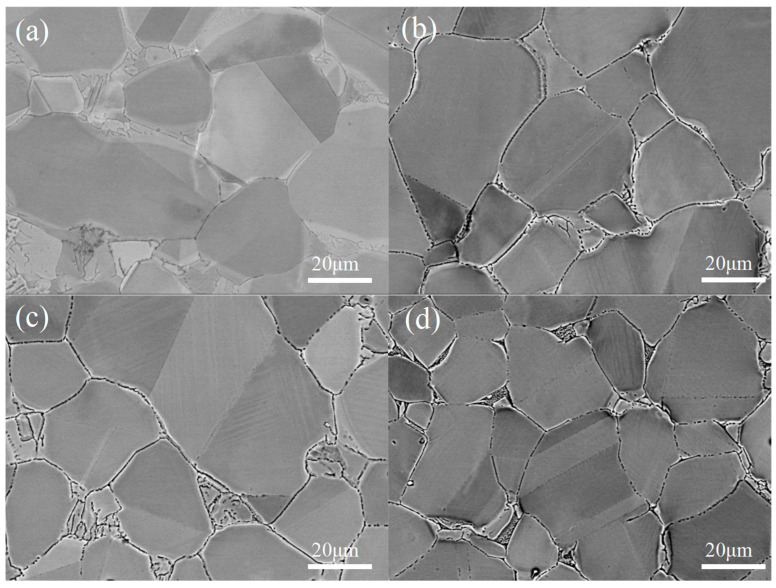
Microstructure characterization of specimens under different process conditions: (**a**) ST; (**b**) AG; (**c**) FC; (**d**) CT.

The metallographic microstructures of different specimens are shown in Figure 5. It was observed from the figure that the grain size of the ST specimen was the largest, reaching 30.1 μm. After aging treatment, the grain size decreased, and the grain size of the AG specimen was 28.0 μm. The grain size of the FC specimen was slightly larger than that of the AG specimen, reaching 29.1 μm, which was attributed to the better insulation effect of the furnace cooling temperature. The grain size of the CT specimen was significantly smaller, at 26.2 μm. By comparison, it was found that after aging treatment, a microstructure similar to twin structure would be formed within the small grains. Compared to the AG specimen, the FC and CT specimens form a more densely packed twin-like structure.

EDS analysis was conducted to characterize the Mn-rich regions formed by different specimens, and the results are shown in Table 3. It was observed that the average Mn content in the Mn-rich region of the specimen after aging treatment was significantly higher than that of the specimen without aging treatment, further indicating that aging treatment promoted the occurrence of spinodal decomposition. By comparison, it was found that the local Mn content of the FC specimen was the highest, reaching 55.76%, followed by the CT and AG specimens. This further indicated that furnace cooling and cryogenic processes had a certain promoting effect on spinodal decomposition.

**Figure 6:**
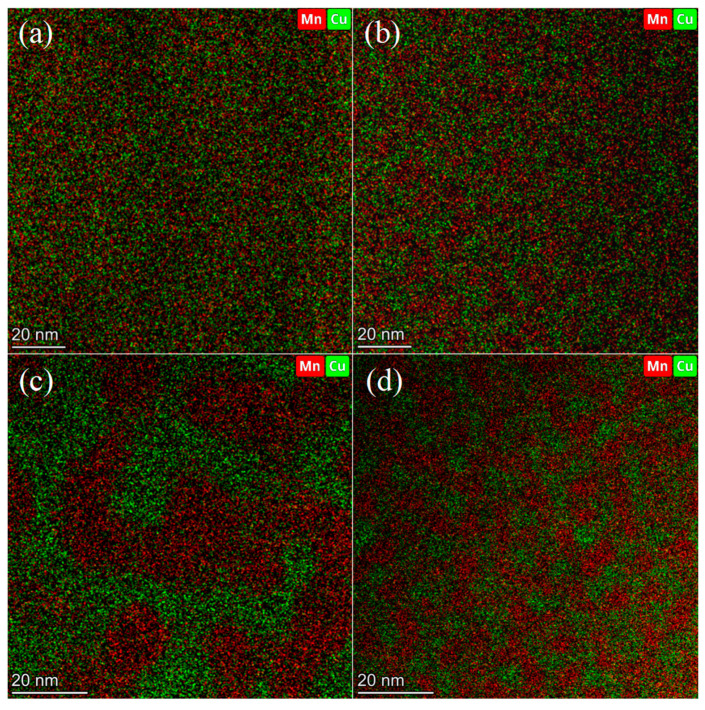
STEM-EDS and SEAD results of specimens under different process conditions: (**a**) ST; (**b**) AG; (**c**) FC; (**d**) CT.

The STEM-EDS images of different specimens are shown in Figure 6. The ST specimen image shows a uniform distribution of Mn and Cu, indicating that no spinodal decomposition had occurred, or the degree of spinodal decomposition was very small. The AG, FC, and CT specimens all formed enrichment zones at the nanoscale of Mn and Cu, indicating that aging treatment promoted the occurrence of spinodal decomposition. As shown in the figure, the size of the nanoscale enrichment zone formed by specimens treated with different processes varies. Among them, the AG specimen formed a nanoscale enrichment zone with a size of about 2–4 nm, the CT specimen formed a slightly larger nanoscale enrichment zone with a size of about 4–6 nm, and the FC specimen formed a significantly larger nanoscale enrichment zone with a size of about 16–20 nm.

**Figure 7:**
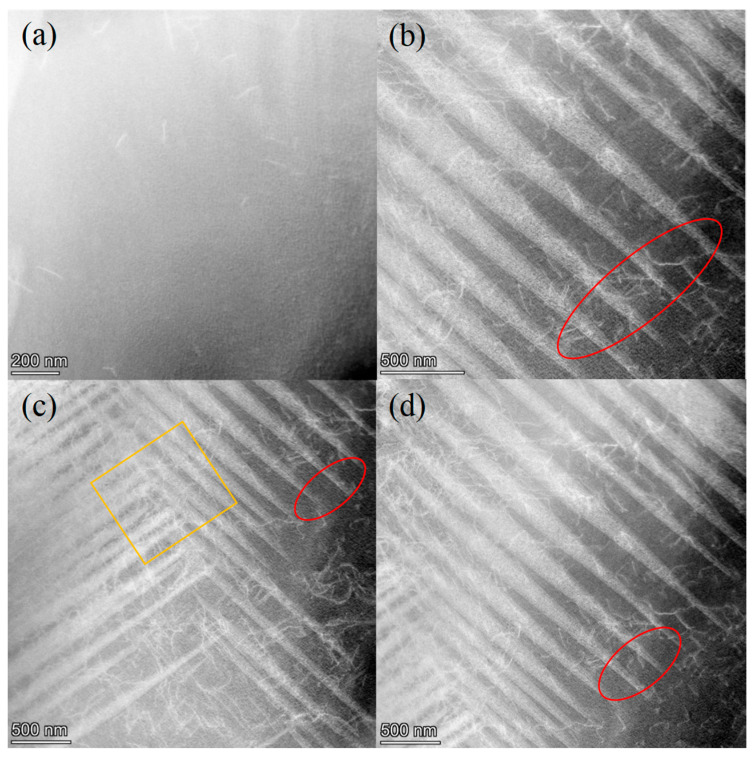
The TEM images of specimens subjected to different process conditions, the red circles indicate the twin tip, and the orange rectangle indicates the twinning with cross distribution: (**a**) ST; (**b**) AG; (**c**) FC; (**d**) CT.

**Figure 8:**
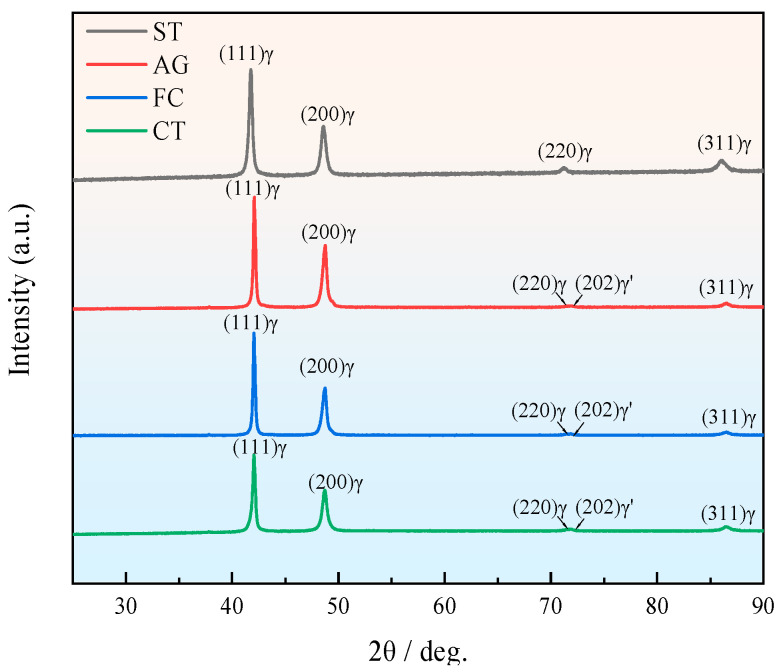
XRD spectra of specimens under different process conditions.

Figure 7 displays TEM images of the specimens under different processing conditions, revealing the twin microstructure. No twin structure was found in the ST specimen, which also confirmed the results in Figure 6 and Figure 8. The number of twin structures found in the other three specimens was significantly increased compared to the ST specimen, and the twinning morphology was more typical, with tips appearing (marked by red circles). In the FC specimen, the number of twinning reached its maximum and exhibited a cross distribution of twinning (marked with orange squares). Compared to the AG specimen, the CT specimen had slightly more twin crystals. After aging treatment, the formation of twinning was promoted. Cryogenic treatment could further increase the twin density, while furnace cooling treatment not only promoted twinning formation, but also caused twinning in different directions, which was also the reason why the FC specimen had the strongest damping capacity.

The XRD patterns of different specimens are shown in Figure 8. It was seen that except for the ST specimen, the (220) γ characteristic peak of the other three specimens showed obvious splitting, resulting in the appearance of the (202) γ characteristic diffraction peak. The splitting of characteristic diffraction peak (220) indicated that the specimens underwent martensitic transformation during the aging process, from the FCC-structured γ phase to the face-centered tetragonal (FCT)-structured γ’ phase [24,25,26].

Based on the data of diffraction peak (220) and Equation (1), the lattice distortion of the AG, FC, and CT specimens was calculated, and the results are shown in Table 4 [27,28].

(1) $$\frac{1}{d^{2}}=\frac{H^{2}+K^{2}}{a^{2}}+\frac{L^{2}}{c^{2}},$$

In Equation (1), d denoted the interplanar spacing, while a and c represented the lattice parameters. Based on Equation (1), the values of a and c could be determined from the positions of the (220) peak and the split (202) peak in the XRD pattern.

According to the calculation results, it was found that the c-axis of the γ’ phase in all three specimens was compressed, resulting in an increase in lattice distortion (a/c − 1), with the CT specimen having the highest distortion. In Mn-Cu alloy, the greater the lattice distortion of the FCT phase, the more martensite formed, which further proved the number of twinnings formed in different specimens in Figure 7.

**Figure 9:**
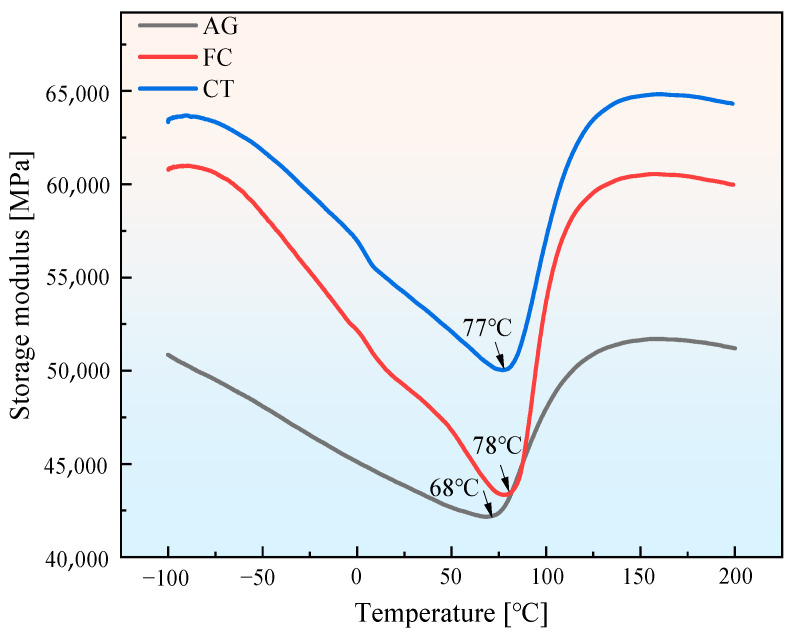
The variation in storage modulus of specimens with temperature.

The more martensite formed in Mn-Cu alloy, the higher the corresponding M_s_ value. Therefore, experiments were conducted on M_s_, and the results are shown in Figure 9. The martensitic transformation in Mn-Cu alloy was accompanied by modulus softening, and M_s_ could be determined by measuring the lowest point of the modulus during the modulus softening process. The FC specimen had the highest M_s_, followed by the CT specimen, and the AG specimen had the lowest. The higher the M_s_ of Mn-Cu alloy with the same composition, the higher the Mn content in the Mn-rich region, indicating that the spinodal decomposition of the alloy was promoted. This also verified the results in Figure 6 and Table 3. Therefore, the cryogenic treatment and furnace cooling process could improve M_s_.

## 4. Discussion

The previous experimental results indicated that there were differences in the microstructure and properties of specimens treated with different processes. The damping capacity of the specimen after solution treatment was the worst, mainly due to the absence of twinning formation. The results in Figure 6 and Table 3 indicate that the specimen did not undergo spinodal decomposition and formed Mn-rich regions, and there was no peak phenomenon on the XRD spectrum, which also proved that phase transition did not occur, and therefore the martensitic twinning did not occur (Figure 7).

The aging treatment processes could improve mechanical properties and damping capacity. The AG specimen underwent spinodal decomposition, resulting in the formation of Mn-rich regions. The solid solution strengthening effect formed after spinodal decomposition promoted the strength. The splitting of the (220) characteristic diffraction peak and the presence of lattice distortion proved the occurrence of martensitic transformation, and the twinning formed was discovered by TEM, which also improved the damping capacity.

By changing the cooling method after aging, it was found that the grain size of the FC specimen further increased after furnace cooling, which had an adverse effect on mechanical properties and reduced strength. However, compared with air cooling after aging, the temperature during furnace cooling decreased slowly, promoting spinodal decomposition. EDS analysis in Table 3 shows that the Mn content in the Mn-rich region was the highest, and M_s_ should also be the highest. The results in Figure 9 prove this, indicating that the FC specimen was more prone to martensitic transformation. TEM images showed a large number of twin crystals with different directions and cross distributions, which was beneficial for the relaxation motion of twin crystals and greatly improved the damping capacity. Its comprehensive performance was better than that of the ST and AG specimens.

For the CT specimen with added cryogenic treatment, the damping capacity was slightly lower than that of the FC specimen, which had been demonstrated in terms of microstructure and structure. Both the degree of spinodal decomposition, lattice distortion caused by martensitic transformation (Figure 8), and the number of twinnings were slightly lower than those of the FC specimen. However, in terms of mechanical properties, that of the CT specimen was significantly higher than that of the FC specimen. Through statistical analysis of metallographic dimensions, it was found that the CT specimen had the smallest grain size and could achieve a fine grain strengthening effect. In addition, dislocations were observed near nanoscale twinning, which was beneficial for improving strength and plasticity. This was related to the promotion of grain refinement and increased in dislocation density through cryogenic treatment.

In summary, after solid solution and aging, Mn-Cu alloys underwent spinodal decomposition, forming Mn-rich regions. In the Mn-rich regions, with the enrichment of Mn element, M_s_ increased, and antiferromagnetic transformation induced martensitic transformation. The coordination and preferred orientation between different regions promoted the formation of twin boundaries. The cryogenic process and furnace cooling process promoted this process, promoted the formation of twin boundaries, and improved damping capacity, and the degree of promotion by furnace cooling was more significant. In addition, cryogenic treatment promoted grain refinement, increased dislocation density, and improved the mechanical properties. The comprehensive index of damping and mechanical properties is an important basis for measuring the applicability of damping materials. The tensile strength of a typical commercial M2052 is greater than 540 MPa, with an elongation of about 32%. When the strain rate is 2 × 10^−4^, the Q^−1^ is 0.025. In contrast, AG and CT specimens have reached the level of commercial M2052 in terms of damping capacity and mechanical properties.

## 5. Conclusions

Comparing the microstructure evolution and properties of specimens under different process conditions, the results were as follows:

1. After solid solution and aging treatment, specimens underwent spinodal decomposition and martensitic transformation, which was beneficial for improving damping capacity. However, specimens that only underwent solid solution treatment were not conducive to damping capacity.
2. Cryogenic treatment and furnace cooling processes promoted spinodal decomposition and martensitic transformation, facilitated the formation of twin boundaries, and improved damping capacity. The degree of promotion by furnace cooling was more significant.
3. Cryogenic treatment promoted grain refinement, increased dislocation density, improved strength, and facilitated the improvement of mechanical properties.

This research will be used to further investigate the damping capacity and mechanical properties of Mn-Cu damping alloys after different heat treatments. For Mn-Cu damping alloys, there is a lack of in-depth research on the quantitative structure–activity relationship between spinodal decomposition, twin boundary structure, and damping performance, which will be a direction for future research.

## Figures and Tables

**Table 1:** Chemical component of Mn-Cu damping alloy (wt.%).

Elements	Cu	Al	Ni	Fe	Mn
Content	39.20	4.25	1.20	3.50	Bal.

**Table 2:** Detailed parameters of heat treatment process.

Specimen	Heat Treatment
ST	Solution treatment + air cooling
AG	Solution treatment + aging treatment + air cooling
FC	Solution treatment + aging treatment + furnace cooling
CT	Solution treatment + cryogenic treatment + aging treatment + air cooling

**Table 3:** Summary of EDS spectroscopy for different heat-treated specimens.

	Mn(wt.%)	Cu(wt.%)	Al(wt.%)	Ni(wt.%)	Fe(wt.%)
ST	48.53	43.49	1.57	2.01	4.40
AG	55.40	36.55	2.80	1.63	3.62
FC	55.76	35.89	2.94	1.68	3.72
CT	55.70	35.30	3.58	1.57	3.85

**Table 4:** Summary of EDS spectroscopy for different heat-treated specimens.

	d_220_	d_202_	a	c	a/c − 1
AG	1.3159	1.3128	3.7219	3.7045	0.0047
FC	1.3175	1.3127	3.7265	3.6994	0.0073
CT	1.3173	1.3128	3.7259	3.7006	0.0068

## Data Availability

The original contributions presented in this study are included in the article. Further inquiries can be directed to the corresponding author.
